# Response to Tahir and Sajid

**DOI:** 10.14309/ctg.0000000000000896

**Published:** 2025-09-08

**Authors:** Krzysztof Dąbkowski, Karolina Skonieczna-Żydecka, Katarzyna Gaweł, Wojciech Marlicz, Piotr Szredzki, Andrzej Białek

**Affiliations:** 1 Department of Gastroenterology, Pomeranian Medical University in Szczecin, Szczecin, Poland;; 2 Department of Biochemical Science, Pomeranian Medical University in Szczecin, Szczecin, Poland;; 3 Endoscopy Unit, Department of Surgery, University of Rzeszów, Rzeszów, Poland.

We would like to express our sincere gratitude to Dr. Shameer Tahir and Dr. Hamza Sajid for their thoughtful and constructive comments on our recently published article (1,2). We welcome the opportunity to respond to their observations, which underscore many of the nuanced considerations in evaluating and comparing minimally invasive approaches for rectal neuroendocrine tumors.

As noted, our meta-analysis included exclusively observational studies, a reflection of the current evidence base in this field. The absence of randomized controlled trials was a limitation we clearly acknowledged in both the Methods and Discussion sections of our article. While this inherently introduces the risk of selection bias—particularly confounding by indication—we sought to mitigate this concern through subgroup analyses and meta-regression. Although variables such as age, tumor size, and follow-up duration were evaluated as potential effect modifiers, none demonstrated statistically significant influence on our primary outcomes. We agree, however, that unmeasured factors—such as operator experience and institutional volume—may have influenced observed differences in outcomes, particularly complication and recurrence rates.

We appreciate the emphasis placed on tumor heterogeneity, specifically regarding grade and invasion depth. Unfortunately, most included studies reported predominantly low-grade (G1) tumors, and the paucity of G2 or G3 lesions limited our ability to perform stratified analyses based on histological grade. Similarly, invasion depth data—ideally assessed by endoscopic ultrasound—were inconsistently reported. We concur that future prospective studies should stratify outcomes by Ki-67 index, invasion depth, and other histopathologic variables, to better support individualized therapeutic decisions.

Tahir and Sajid also raise an important point regarding differential follow-up times. Indeed, the median follow-up period was significantly longer in the transanal endoscopic microsurgery cohort (48.2 months) compared with submucosal dissection (28 months), and this was explicitly discussed in our manuscript. We highlighted this as a likely explanation for the paradoxical finding of higher recurrence rates after transanal endoscopic microsurgery, despite its higher R0 resection rate. Rather than reflecting inferior oncologic control, the higher recurrence rate may in part represent a detection bias associated with prolonged surveillance. This underlines the broader need for standardized, long-term follow-up protocols across all treatment modalities for rectal neuroendocrine tumors.

We strongly agree that functional outcomes—including continence, sphincter preservation, and quality of life—are critical to evaluating treatment efficacy from a patient-centered perspective. However, these outcomes were rarely reported in the studies we reviewed and therefore could not be included in our quantitative synthesis. We regard this as a significant evidence gap and support the call for future studies to prioritize these parameters in both design and reporting.

Regarding procedural economics, we share the view that comparative cost-effectiveness is an important component of health policy and clinical decision making. Although submucosal dissection is generally less resource-intensive and does not require general anesthesia or an operating theater, formal cost analyses were absent in the studies we included. This is a critical area for future investigation, particularly in resource-constrained settings.

Last, we acknowledge the limitations of using funnel plots and Egger's tests to assess publication bias in outcomes with a limited number of studies. While we did apply these methods, we also interpreted the results with caution and disclosed the limitations of these techniques within the manuscript.

In conclusion, we thank Drs. Tahir and Sajid for their insightful engagement with our work. Their letter reinforces several important methodological and clinical points and highlights key areas for future research, including standardized outcome reporting, biomarker-informed treatment stratification, and incorporation of patient-reported outcomes and economic evaluations. We remain committed to advancing high-quality, translationally relevant evidence in the care of patients with rectal neuroendocrine tumors.

## CONFLICTS OF INTEREST

**Guarantor of the article:** Krzysztof Dąbkowski, PhD.

**Specific author contributions:** All authors approved the final version and drafted the response.

**Financial support:** Original study was supported by Pomeranian Medical University.

**Potential competing interests:** None to report.
